# Toxicokinetics of Deoxynivalenol in Dezhou Male Donkeys after Oral Administration

**DOI:** 10.3390/toxins15070426

**Published:** 2023-06-30

**Authors:** Ruifen Kang, Honglei Qu, Yanxin Guo, Chuanliang Ji, Jie Cheng, Yantao Wang, Shimeng Huang, Lihong Zhao, Cheng Ji, Qiugang Ma

**Affiliations:** 1 State Key Laboratory of Animal Nutrition, College of Animal Science and Technology, China Agricultural University, Beijing 100193, China; 2 National Engineering Research Center for Gelatin-Based Traditional Chinese Medicine, Dong-E-E-Jiao Co., Ltd., Liaocheng 252201, China

**Keywords:** Kinetics, toxicity, mycotoxins, vomitoxin, mammals

## Abstract

Deoxynivalenol (DON) is detected in different types of foods and feeds, inducing toxicity in humans and animals. After entering the organism, DON first appears in the plasma; then, it is rapidly absorbed and distributed in various organs and tends to accumulate in the body to exert its toxic effects. This study was performed to investigate the toxicokinetics of DON on Dezhou male donkeys after a single oral dose of 500 μg/kg·BW (body weight). The plasma of donkeys was collected at 0, 5, 10, 15, 20, 30, 45 min, 1, 1.5, 2, 2.5, 3, 3.5, 4, 4.5, 6, 9, 12, 24, 48, 72, 96 and 120 h after administration, and the feces and urine were collected at 0 h and at 6 h intervals up to 24 h, followed by 4 h intervals up to 120 h. The concentrations of DON in plasma, urine and feces were determined by HPLC. The peak concentration of DON in plasma was 174.30 μg/L, which occurred at 1.07 h after oral gavage. The recovery of unchanged DON in urine and feces amounted to 19.98% and 6.74%, respectively. Overall, DON was rapidly absorbed and slowly eliminated in donkeys within 120 h following a single oral dose, which can lead to DON accumulation in the body if ingested for a long time.

## 1. Introduction

Deoxynivalenol (DON) belongs to group B trichothecenes, is a kind of secondary metabolites produced by *Fusarium graminearum* and *Fusarium culmorum* [1] and is widely present in various cereals, including corn, soybean, wheat and barley [2,3]. In a large-scale investigation of 74,821 food samples worldwide during the decades 2008–2017, the detection rate of DON reached up to 64% [4]. DON was also the most contaminated mycotoxin in different provinces in China during 2016–2017 (n = 1569), with a contamination rate of 74.5% and an average detection concentration of 450–4381.5 μg/kg [5]. The positive detection rate of DON in wheat (n = 579) and maize (n = 606) samples were 100% and 99.83%, respectively, with an average detection concentration of 165.87 μg/kg and 175.30 μg/kg, respectively [6]. Moreover, DON has epoxy groups with resistance to abrasion and high temperatures and could be stable in low-pH and high-temperature environments [7]. Under laboratory conditions, DON could be stored for a long time to maintain the same toxicity. Moreover, the toxicity remains stable under food or feed processing. Thus, DON could easily pass through the food chain, causing adverse effects on the health of humans and animals [7,8]. Different animals have different sensitivities to DON, and mammals are more sensitive. It has been reported that DON could cause intestinal inflammation in piglets after the administration of 3 mg DON/kg feed for 35 days [9]. DON also disrupts respiratory epithelial morphology, further affecting the epithelial integrity of horse [10]. In addition, the treatment of mice with 4.70 mg/kg DON for 28 days significantly reduced daily weight gain and increased serum urea nitrogen and tumor necrosis factor-α [11]. Similarly, feeding pigs DON-containing feed for 18 days significantly reduced daily weight gain and increased serum IgG and GSH-Px levels [12]. Four weeks after mice were given 2.4 mg DON/kg BW, the percentage of abnormal spermatozoa increased, sperm motility decreased, and testicular morphology was severely disrupted. In addition, the levels of ROS and MDA increased, and the levels of SOD and GSH decreased, indicating that DON treatment induced oxidative stress [13]. These studies suggest that DON is not only widely presented in foods or feeds but also causes toxic effects in animals, including enterotoxicity, immunotoxicity and reproductive toxicity.

After entering the organism, DON first appears in the plasma. Then, DON is rapidly absorbed and distributed in various organs and tends to accumulate in the intestine, which is the key target organ for DON to exert its toxic effects. Different animals vary in their susceptibilities to DON in the order of pigs, mice, rats and poultry, which may be related to the different in absorption, distribution, metabolism and excretion of DON in animals [14]. Meanwhile, the toxicokinetics of DON have been studied on different kinds of animals, including pigs [15], chickens [16], mice [17], rats [18] and fish [19]. The time to reach the maximum plasma DON concentration (T_max_) after oral administration was about 0.08–0.5 h in mice [20,21], 2 h in chicken [22] and 10 min in rats [23]. However, the T_max_ in pigs was about 1.5–4.1 h [24], which was much slower than the other animals. The elimination half-life (T_1/2_) of DON in pigs was in the range of 1.5–5.3 h [23,25], which was much slower than mice [17,21], rats [23] and chickens [26]. Therefore, the toxicokinetics of DON are correlated with the susceptibility of animals to the toxin, and it is necessary to assess the toxic effects of the toxin on animals. 

The donkey has a high economic value, not only as a companion animal and a service domestic animal but also as an important source of food and medicine, including donkey meat and donkey-hide gelatin [27]. However, to our knowledge, there have been few reports on the toxicokinetics of DON in donkeys. Therefore, the aim of this study was to investigate the toxicokinetics of DON in donkeys after receiving a single gavage of DON-containing material.

## 2. Results

### 2.1. Validation Parameters

**Figure 1:**
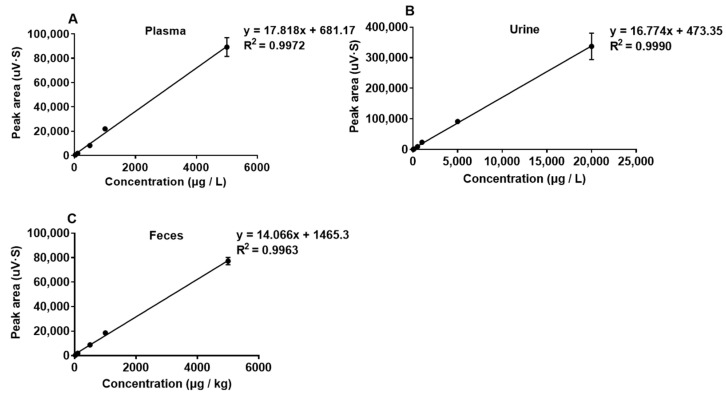
The calibration curves for spiked samples (25, 100, 500, 1000, 5000, 20,000 μg/L or μg/kg) of deoxynivalenol (DON) in plasma (**A**), urine (**B**) and feces (**C**), n = 3.

The calibration curves showed a linear trend in the range of 25–20,000 μg/L (Figure 1). A good coefficient of determination (R2 ≥ 0.9963) was obtained for DON in plasma, feces and urine, respectively. For DON, the limit of detection (LOD) and the limit of quantification (LOQ) was 25 μg/L and 100 μg/L, respectively, in the plasma, urine and feces of donkeys (Table 1). All values showed that the sensitivity of the method met the requirements of the determination of DON in plasma, urine and feces. The recovery of DON was assessed at five levels (25, 100, 500, 1000 and 5000 μg/L or μg/kg) in plasma and feces, and at six levels (25, 100, 500, 1000, 5000 and 20,000 μg/L) in urine, with mean values of 86.56%, 81.50% and 87.94%, respectively, in plasma, feces and urine (Table 2).

### 2.2. Plasma Toxicokinetics Parameters of DON 

The toxin was absorbed into the blood circulation after the oral gavage, and the concentrations of DON in plasma were determined. The plasma levels of DON reached a peak concentration (C_max_) at 1.07 ± 0.74 h (T_max_) after oral dosing. The elimination of half the amount of DON in donkeys took 5.81 ± 0.77 h (Table 3). 

### 2.3. Plasma Concentration of DON

**Figure 2:**
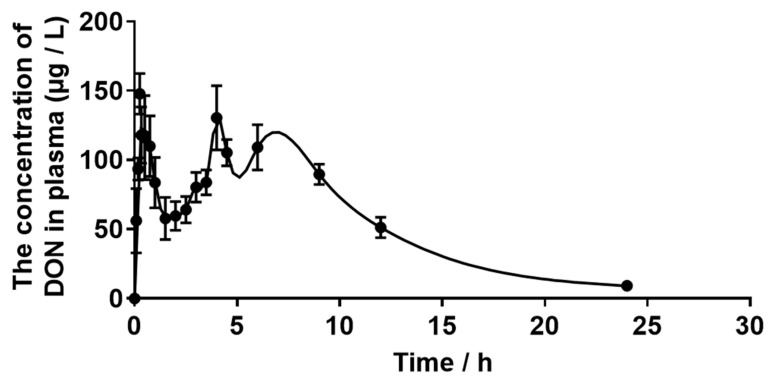
Plot of mean plasma deoxynivalenol (DON) concentrations vs. time (0, 5, 10, 15, 20, 30, 45 min, 1, 1.5, 2, 2.5, 3, 3.5, 4, 4.5, 6, 9, 12 and 24 h) in donkeys after a single oral administration of 500 μg/kg·BW, n = 5.

As shown in Figure 2, the plot revealed that the DON was first found in plasma 5 min after the gavage, and the concentration of DON increased over time until the maximum concentration was reached at 1.07 ± 0.74 h after oral dosing. DON could not be detected 24 h after administration.

### 2.4. Recovery of DON Eliminated in Urine and Feces

**Figure 3:**
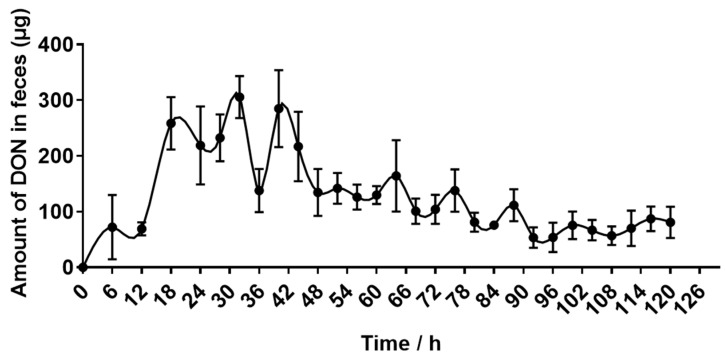
The average excretion of deoxynivalenol (DON) in the feces of donkeys after a single oral administration of 500 μg/kg·BW, n = 5.

The DON-containing feces were first eliminated 6 h after oral administration, and the elimination increased rapidly between 12 h and 32 h. Then, the amount of eliminated DON began to decrease until low levels of DON could be detected at 120 h (Figure 3).

**Figure 4:**
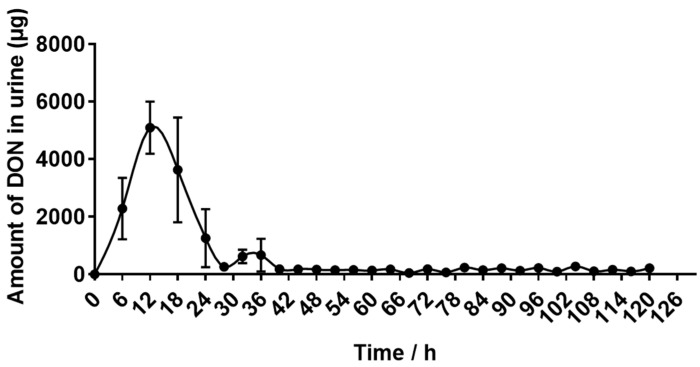
The average excretion of deoxynivalenol (DON) in the urine of donkeys after a single oral administration of 500 μg/kg·BW, n = 5.

As shown in Figure 4, DON-containing urine were first eliminated 6 h after oral administration, and the elimination increased rapidly between 6 h and 12 h. Then, the amount of eliminated DON began to decrease until low levels of DON could be detected at 40 h.

The excretion of feces and urine are two main routes for removing toxins in animals. As shown in Table 4, donkeys consumed a total of 65.50 ± 0.52 mg of DON, of which 6.74 ± 0.51% was excreted in the feces and 19.98 ± 2.46% in the urine. The absorption rate was 93.26 ± 0.51%. These values suggest that donkeys have a high absorption rate of DON, and urine is the main route of DON excretion. 

## 3. Discussion

The toxicokinetics of DON varies in different animals due to variances in the processes of absorption, distribution, metabolism and clearance of DON. Donkeys were used as the subjects in the present study for their importance in providing food and medicine. Moreover, donkeys are indispensable for people’s daily work, including the transportation of goods, plowing and travel. However, there have been no reports on the toxicokinetics of DON in donkeys following oral administration. Therefore, this study was conducted to investigate the toxicokinetics of DON after oral administration to donkeys.

Due to the relatively large size of donkeys and the high price of pure toxin, the DON-containing material used in this experiment was obtained by the fermentation of *Fusarium graminearum 37687*, whose ability to produce DON toxin has been verified in previous work [12]. DON production is mainly influenced by temperature and humidity. Studies have shown that low temperatures and high humidity tend to delay crop harvest and allow mold to persist on the crop, resulting in high levels of DON [1,28]. Therefore, in the present study, we strictly controlled the temperature at 25 °C, and the high humidity environment was ensured by soaking the corn for 12 h in advance, adding extra water to corn at a ratio of 1:4 during inoculation and placing sterile water in the incubator during fermentation. We found that under the controlled temperature and humidity of the present experiment, the toxin-producing bacteria could grow well and have a high capacity for DON toxin production. However, we also detected a small amount of other toxins in the moldy feed, such as 15ADON, 3ADON and ZEA. It is worth noting that even low doses of toxins can cause adverse effects on animals [29]. In addition, whether these toxins interfered with the metabolism of DON was also unclear, and further studies should be performed in the future.

DON is rapidly absorbed by digestive tract of animals. Studies have shown that after the oral intake of DON, up to 52.7–100%, 47.3% and 5.6–19.3% of DON was absorbed rapidly in the gastrointestinal tract of pigs [24], rats [23] and chicken [16,30], respectively. After the absorption, the maximum levels of DON first appeared in plasma. The T_max_ was reported to be 3.5 h for Norwegian crossbred pigs fed 0.125 mg DON/kg BW [31], and after pigs were fed a diet at a concentration of 4.2 mg DON/kg, the T_max_ was 4.1 h [15]. After a single oral dose of 2.2 mg ^14^C-DON to hens, the maximum radioactivity value in plasma was detected after 2 h [32]. The T_max_ was reported to be 5–30 min for mice after the oral administration of 25 mg DON/kg BW [17,33]. In addition, the T_max_ was 10 min after administration of 0.1 mg DON/kg BW in rats [23]. These results suggest that the T_max_ in pigs is much longer than that in chicken, mice and rats. In addition, the half-life of DON elimination was 0.34–0.68 h in mice [17,21], 0.46 h in rats [23] and 0.6–1.3 h in chickens [16,26], all of which were faster than that of pigs [23,25]. DON is highly absorbed and slowly eliminated in pigs, which may be one of the reasons why pigs are more sensitive to DON than other animals. In the present study, we found that the concentrations of DON in plasma reached the maximum at 1.07 ± 0.74 h after oral administration, which was shorter than pigs and chickens but longer than mice and rats. However, the elimination half-life in donkeys was 5.81 ± 0.77 h, which was longer than all of the abovementioned animals. The results suggest that DON is eliminated slowly by donkeys and tends to accumulate in the body. We could speculate that the donkeys may be more sensitive to DON than pigs, but this needs more data for verification.

The excretion of feces and urine are two main routes for removing toxin in animals. However, animals have different strategies for excreting toxins, which leads to differences in excretion patterns. The main metabolic pathways of DON in animals are gut microbial transformation and phase II metabolism. In other words, DON is absorbed by digestive tract into the plasma and carried to the liver, where it is efficiently metabolized by liver enzymes and excreted in the urine. The unabsorbed DON is transformed by microorganisms in the intestine and excreted in the feces in the forms of DON and de-epoxy-DON (DOM-1) [14]. However, the ability of microorganisms to convert DON to DOM-1 is very limited, and studies have shown that DOM-1 could not be detected in the feces of pigs [34], while it could be detected in the feces of rats [35], suggesting that microbial transformation may not be the main excretion route in pigs, but it is the main excretion route in rats. In addition, it has been reported that for the oral administration of 2.5 mg DON/kg BW to rats, 40–48% and 28–33% was excreted through the feces and urine, respectively [36]. Similar to rats, 61.3% and 33.2% of DON was excreted by feces and urine after administration of 2.5 mg DON/kg BW to chickens [36]. However, after the administration of 1 mg DON/kg BW to mice, 49–86% of DON was discovered in urine, and only 1.2–8.3% was found in feces [37]. Similar to mice, 49.7% and 2.5% of DON was excreted by urine and feces, respectively, after the administration of 5.96 mg DON/d to pigs [25]. These studies showed that pigs and mice excreted more DON from urine than from feces, while rats and chickens excreted more DON from feces than from urine, suggesting that phase II biotransformation might play a major role in DON elimination in pigs and mice, while gut microbiota plays an important role in the elimination of DON in rats and chickens. In the present study, after oral administration with 0.5 mg DON/kg BW to donkeys, in 120 h, 19.98% of DON was excreted by urine, and only 6.74% was excreted by feces. The results suggest that DON is mainly excreted by urine in donkeys. However, in donkeys, the amount of DON excreted in the urine was smaller than in pigs and mice, which may be related to the size of the animals, toxin dosage and route of exposure.

## 4. Conclusions

The present study suggests that DON has a high absorption rate following a single oral dose of 500 μg DON/kg BW in male donkeys; however, elimination was slow, indicating that DON tended to accumulate in the donkeys. In addition, DON was mainly excreted through urine in donkeys, accounting for 19.98% of the total intake, and only 6.74% was found in the feces. However, the biotransformation of DON in the guts and liver of donkeys is still unclear, and further studies on the toxic kinetics of modified DON, such as 3-Acetyldeoxynivalenol (3ADON) and 15-Acetyldeoxynivalenol (15ADON), should be carried out in the future.

## 5. Materials and Methods

### 5.1. Mycotoxins

The DON standard solution was purchased from Pribolab Biological Engineering Co. Ltd. (Qingdao, China). The DON-containing material used in this experiment were produced by *Fusarium graminearum 37687*, which was provided by the Agricultural Culture Collection of China (ACCC).

The fungi were activated on Potato-dextrose Agar (PDA) (Guangdong Huankai Microbial Sci.&Tech.CO., Ltd., Guangzhou, China) at 25 °C for 7 days, and then inoculated in corn at 25 °C for 3 weeks to produce DON. Before inoculation, the corn was washed, soaked in water for 12 h and then autoclaved for 30 min at 121 °C. After cooling down, the fungi were inoculated into the corn; then, sterile water was added in the ratio of corn:water = 4:1 and placed in the incubator for 21 days. A container with sterile water was placed into the incubator to adjust the humidity during the incubation period. After 21 days, the moldy corn was dried in an oven at 40 °C and then crushed into powder to determine the toxin content. Deoxynivalenol was extracted from the homogenized culture material by adding acetonitrile/water (10:90 *v*/*v*) ultrasonically for 40 min. The extract was then filtered using a Whatman filter (Whatman, Maidstone, Kent, UK) to determine the mycotoxin content. The levels of zearalenone (ZEN), aflatoxins B 1(AFB1) and Ochratoxin A (OTA) in the moldy corn were determined using HPLC [38]. The concentration of ZEA was 258.75 μg/kg, and the AFB1 and OTA were not detected in the moldy corn. In addition, the levels of DON, 15-Acetyldeoxynivalenol (15ADON) and 3-Acetyldeoxynivalenol (3ADON) in the moldy corn were determined using LC-MS/MS [39]. The concentrations of DON, 15ADON and 3ADON were 124.07 mg/kg, 6.244 mg/kg and 11.37 mg/kg, respectively (Figure S1).

### 5.2. Animals and Treatment

The study was allowed by the Laboratory Animal Welfare and Animal Experimental Ethical Committee of China Agricultural University (No. AW30103202-1-1), approved on 3 January 2023. Five 9-month-old male donkeys were selected and housed individually in metabolic cages. The donkeys were allowed to adapt to their surroundings for five days and fast overnight before the experiment; then, the body weight (BW) of donkeys was measured (131.00 ± 1.05 kg). The samples of blank blood, urine and feces were collected at 4 h prior to the start of the experiment. The culture material of *Fusarium graminearum 37687* was dried in an oven at 40 °C and then crushed into powder. At the start of the experiment, the powder was dissolved in sterile water and orally administered through an esophageal tube in a single dose of 500 μg DON/kg BW. The animals had free access to feeds and water during the experimental period. The AFB1, OTA, 15ADON and 3ADON were not detected in both the forage feed and concentrate feed. The levels of DON and ZEA were 417.72 and 139.48 μg/kg and 408.79 and 255.29 μg/kg in the concentrate feed and forage feed, respectively.

### 5.3. Blood, Plasma, Feces and Urine Collection

The blood samples (post-hepatic) were collected from the jugular vein of donkeys and placed in heparin anticoagulation tubes before administration (0 min) and 5, 10, 15, 20, 30, 45 min, 1, 1.5, 2, 2.5, 3, 3.5, 4, 4.5, 6, 9, 12, 24, 48, 72, 96 and 120 h after administration. The blood samples were centrifuged at 3000 rpm for 20 min at 4 °C to obtain plasma and were stored at −20 °C for the analysis of DON levels. The urine and feces were collected before administration (0 h) and 6, 12, 18, 24, 28, 32, 36, 40, 44, 48, 52, 56, 60, 64, 68, 72, 76, 80, 84, 88, 92, 96, 100, 104, 108, 112, 116 and 120 h after administration for the analysis of DON concentration.

### 5.4. Standard Solutions

First, 10 mg of DON was dissolved in 2 mL of methanol (Dikma Technologies Inc., Beijing, China) to obtain a 5 mg/mL DON stock solution. The stock solution was diluted with acetonitrile/water (50/50, *v*/*v*) to obtain different levels of DON working solutions (0.25, 1, 5, 10, 50 and 200 μg/mL). Then, 90 μL of blank samples of plasma and urine were taken, and 10 μL of working solutions were added to obtain spiked samples with five concentrations of DON in the range of 25–5000 μg/L for plasma and six concentrations of DON in the range of 25–20,000 μg/L for urine. In addition, 100 μL of working solutions were added to 1 g of blank fecal samples to obtain spiked samples with five concentrations ranging from 25–5000 μg/L.

### 5.5. Sample Pretreatment

In total, 100 μL of thawed samples of plasma and urine were added to centrifuge tubes (2 mL); then, 400 μL of ethyl acetate (Dikma Technologies Inc., Beijing, China) was added, vortexed for 5 min and centrifuged for 5 min at 12,000 rpm. The samples of feces were dried and homogenized, and 1.0 g was transferred to a 10 mL centrifuge tube. A volume of 4 mL of ethyl acetate was added, vortexed for 5 min and centrifuged for 5 min at 12,000 rpm. The ethyl acetate extraction was repeated twice, and the supernatant was combined and evaporated in a vial at 40 °C. Then, the samples of plasma and urine were reconstituted in 100 μL of methanol/water (15/85, *v*/*v*), and feces were reconstituted in 1 mL of methanol/water (15/85, *v*/*v*). All samples were vortexed for 1 min and then filtered using a 0.22 μm filter for analysis by HPLC.

### 5.6. HPLC Method Validation

The method was validated in plasma, urine and feces on the basis of linearity, sensitivity and recovery. The calibration curves were prepared by adding different concentrations of DON to blank matrices (plasma, urine and feces). Sensitivity was calculated by LOD and LOQ. The signal-to-noise ratio (S/N) was ≥3 for LOD and S/N≥ 10 for LOQ. Recovery was calculated by the ratio of the peak areas of different concentrations of DON obtained from spiked samples of plasma, urine and feces to the areas of the corresponding standard working solutions of DON.

### 5.7. Quantification of DON by HPLC

Quantification of DON was achieved using an HPLC system equipped with an ultraviolet monitor (SPD-20A, Shimadzu, Kyoto, Japan) at a wavelength of 218 nm. The mobile phase consisted of methanol and water (15:85, *v*/*v*) at a flow rate of 1.0 mL/min and an injection volume of 20 μL.

### 5.8. Data Standardization

As the feeds contained natural DON, the DON concentrations in plasma, urine and feces prior to gavage (C_0_) were subtracted from the concentrations of DON in plasma, urine and feces at different time points (C_T_S) using the formula “C_T_S-C_0_” to standardize the data before the statistical analysis.

### 5.9. Statistical Analysis

The statistical analysis was based on standardized concentrations of DON in plasma, urine and feces. The non-compartmental model method in WinNonlin 5.2.1 software (Certara, Inc., Princeton, NJ, USA) was used to calculate the plasma toxicokinetic parameters of DON. Meanwhile, the mean concentrations of DON in plasma at different times were used to plot the DON concentration-time curve; the mean excretions of DON in urine and feces at different times were used to plot the DON excretion–time curves. Data are expressed as mean ± SEM. The figures were plotted using GraphPad Prism version 7.01 (GraphPad Software, Inc., San Diego, CA, USA).

## Figures and Tables

**Table 1:** The calibration curves of deoxynivalenol in plasma, feces and urine.

Matrix	Slope	R^2^	Range (μg/L)/ (μg/kg)	Sensitivity (μg/L)/(μg/kg)	
				LOD	LOQ
Plasma	17.818	0.9972	25–5000	25	100
Feces	14.066	0.9963	25–5000	25	100
Urine	16.774	0.9990	25–20,000	25	100

μg/L refers to the values of deoxynivalenol (DON) in plasma and urine, μg/kg refers to the values of DON in feces, n = 3 of each concentration. LOD: limit of detection, LOQ: limit of quantification.

**Table 2:** The recovery of deoxynivalenol for plasma, feces and urine.

Spike Level (μg/L)/(μg/kg)	Average Recovery (%)		
	Plasma	Feces	Urine
25	86.91	78.16	87.58
100	84.85	88.84	75.02
500	83.17	88.63	93.04
1000	87.03	73.30	92.54
5000	90.82	78.58	93.18
20,000	—	—	86.25

n = 3 of each concentration.

**Table 3:** Plasma toxicokinetic parameters following the oral gavage of deoxynivalenol in donkeys.

Parameters	Value
Body weight (kg)	131.00 ± 1.05
Single oral dose of DON (μg·kg·BW^−1^)	500
T_max_ (h)	1.07 ± 0.74
C_max_ (μg·L^−1^)	174.30 ± 14.27
T_1/2_Elim (h)	5.81 ± 0.77
AUC (μg·L^−1^·h)	1540 ± 195.50
Cl (L·kg·BW^−1^·h^−1^)	0.35 ± 0.057
Vd (L·kg·BW^−1^)	2.82 ± 0.35

T_max_: time of occurrence of maxima concentration of deoxynivalenol (DON) in plasma, C_max_: concentration maxima of DON in plasma, T_1/2_Elim: terminal elimination half-life, AUC: area under plasma concentration-time curve, Cl: total plasma clearance, Vd: volume of distribution.

**Table 4:** Deoxynivalenol excretion in urine and feces following oral administration of 500 μg/kg·BW in donkeys.

Parameters	Value
DON intake (mg)	65.50 ± 0.52
DON excretion through feces (mg)	4.42 ± 0.35
DON excretion through feces (%)	6.74 ± 0.51
DON excretion through urine (mg)	13.12 ± 1.69
DON excretion through urine (%)	19.98 ± 2.46
Absorption rate (%)	93.26 ± 0.51

Deoxynivalenol (DON) excretion through feces (%) = DON excretion through feces (mg)/DON intake (mg) × 100. DON excretion through urine (%) = DON excretion through urine (mg)/DON intake (mg) × 100. Absorption rate (%) = (DON intake (mg) − DON excretion through feces (mg))/DON intake (mg) × 100.

## Data Availability

The data presented in this study are available on request from the corresponding author.

## Supplementary Materials

The following supporting information can be downloaded at: https://www.mdpi.com/article/10.3390/toxins15070426/s1, Figure S1: MRM of deoxynivalenol (DON), 15-Acetyldeoxynivalenol (15ADON) and 3-Acetyl deoxynivalenol (3ADON) in the moldy corn.
