# Supplementary material for: Toxicokinetics of Deoxynivalenol in Dezhou Male Donkeys after Oral Administration
Source: Toxins (Basel). 2023 Jun 30;15(7):426. doi: 10.3390/toxins15070426 (PMC10467147; doi:10.3390/toxins15070426)
Supplement: Supplementary file 1 [file toxins-15-00426-s001.zip › toxins-2282868-supplementary.pdf]

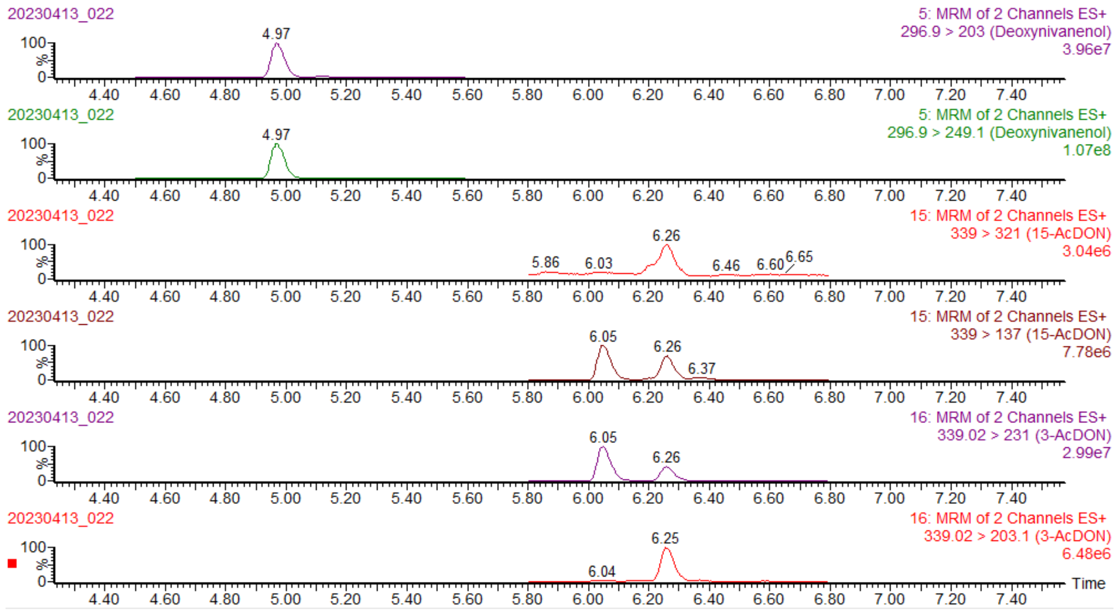

Figure S1. MRM of deoxynivalenol (DON), 15-Acetyldeoxynivalenol (15ADON) and 3-Acetyl deoxynivalenol (3ADON) in the moldy corn.
